# Dynamical dispersion engineering in coupled vertical cavities employing a high-contrast grating

**DOI:** 10.1038/s41598-017-02394-9

**Published:** 2017-05-18

**Authors:** Alireza Taghizadeh, Il-Sug Chung

**Affiliations:** DTU Fotonik, Department of Photonics Engineering, Technical University of Denmark, Building 343, DK-2800 Kgs. Lyngby, Denmark

## Abstract

Photon’s effective mass is an important parameter of an optical cavity mode, which determines the strength of light-matter interaction. Here, we propose a novel method for controlling the photon’s effective mass by using coupled photonic cavities and designing the angular dependence of the coupling strength. This can be implemented by employing a high-contrast grating (HCG) as the coupling reflector in a system of two coupled vertical cavities, and engineering both the HCG reflection phase and amplitude response. Several examples of HCG-based coupled cavities with novel features are discussed, including a case capable of dynamically controlling the photon’s effective mass to a large extent while keeping the resonance frequency same. We believe that full-control and dynamical-tuning of the photon’s effective mass may enable new possibilities for cavity quantum electrodynamics studies or conventional/polariton laser applications. For instance, one can dynamically control the condensate formation in polariton lasers by modifying the polariton mass.

## Introduction

When several optical cavities are coupled to each other, they can display a number of interesting physical phenomena, such as miniband formation^1^, heavy photons^2^, coupled-cavity quantum electrodynamics^3^, slow-light^4, 5^, and parity-time symmetry breaking^6^. From an application point of view, a coupled cavity system can improve the performance of an optical device considerably, such as increasing the laser modulation speed^7^ or its differential quantum efficiency^8^, suppressing higher-order modes of a micro-cavity laser^9^, and enhancing the photodetector light absorption^10^. The coupled cavity systems are implemented in various platforms, including photonic crystals^1, 8, 11^, microrings^6, 9^, or vertical cavity structures (VCSs)^10, 12^. In particular, the vertical cavity platform is advantageous for applications requiring low loss or spatially-extended matter-light coupling, since the active materials can be integrated into the VCS without loss penalty^13^. A coupled VCS comprises three mirrors, which are usually implemented as distributed Bragg reflectors (DBRs)^12^.

**Figure 1:**
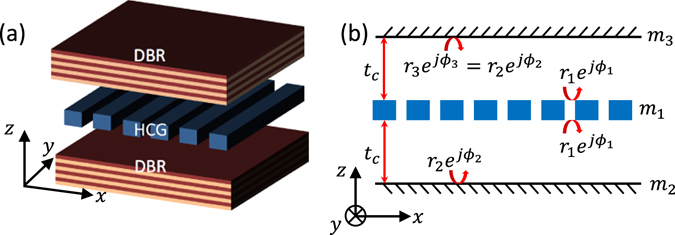
(**a**) Schematic view of a system of two coupled vertical cavities realized by a HCG reflector (coupling mirror *m*_1_) and two conventional DBRs (outer mirrors *m*_2_ and *m*_3_). (**b**) The schematic cross-sectional view of (**a**), where *m*_2_ and *m*_3_ are shown as simple reflectors, and assumed to be similar. The mirror reflection amplitudes, *r*_*i*_ (real and positive), phases, *ϕ*_*i*_ (0–2 *π*), and nominal cavity length, *t*_*c*_ are defined as shown in figure.

Here, we propose a novel method to engineer the dispersion of cavity modes in a coupled VCS by employing a high-contrast grating (HCG) reflector^14–18^ as a coupling mirror between two cavities, as illustrated in Fig. 1(a). Engineering the dispersion property of a photonic system, i.e. the relationship between the mode frequency and wavevector *ω*–*k*, is a fundamental approach to manipulate the light behavior. For instance, in photonic crystal waveguides, the dispersion engineering of waveguide modes enables us to slow down the light^19, 20^, or to form an ultra-high Q-factor resonator^21^. In polariton lasers, which recently have emerged as a new type of light-source with ultra-low threshold lasing^22^ or other interesting phenomena including super-fluidity and bi-stability^22^, one can control the dynamics and condensate formation by engineering the mode dispersion^23^. Furthermore, the light-matter interaction can be modified by engineering the dispersion property, since the optical density of states (DOS) depends on the dispersion. For instance, the spontaneous emission rate of an emitter in the optical cavity can be enhanced through the Purcell factor^24^. The dispersion in a VCS represents the dependence of the mode resonance frequency on the in-plane wavevector, which has a parabolic characteristic with its curvature interpreted as the photon’s effective mass^25–27^. As will be discussed, in HCG-based coupled VCSs, the angular dependence of reflection amplitude and phase of HCG allows to fully control and dynamically tune the dispersion of cavity modes, which result in novel features for this structure.

This paper is organized as follows. First, analytical expressions for the mode resonance frequencies and dispersion curvature in a single VCS and a system of two coupled vertical cavities are derived. While, the dispersion calculations and discussions in the paper are performed for the *x*-direction (c.f. Fig. 1), i.e. perpendicular to the grating bars, similar result can be obtained for the *y*-direction, i.e. along the grating bars. It is shown that three types of dispersions are possible for the coupled system including an interesting dispersion with a characteristic Dirac-cone like point. Then, the possibility of designing a mode with large dispersion curvature while achieving large Q-factor, is shown with numerical simulation. Finally, a novel coupled VCS based on the recently-proposed hybrid grating (HG)^16, 28^ is suggested and investigated, which possesses more feasible fabrication process. It is shown that by mechanically moving one of the mirrors in this structure, it is possible to hugely modify the photon’s effective mass without altering its energy considerably. This possibility of tuning photon’s effective mass dynamically may be valuable in various applications. For instance, the effective mass of the polariton quasi-particle in a polariton laser can be varied during an experiment, which would influence the thermalization of polaritons or their transport properties^13^.

## Results

### Single vertical cavity dispersion

The mode resonance frequencies *ω* of a single VCS, formed just by the two mirrors *m*_1_ and *m*_2_ [c.f. Fig. 1(b)], are the frequencies where transmissivity becomes maximum. They correspond to the constructive interference condition for the round-trip phase (see Supplementary Information for details), $\psi \triangleq {\varphi }_{1}(\omega ,{k}_{x})+{\varphi }_{2}(\omega ,{k}_{x})-2{k}_{z}{t}_{c}=2m\pi$. Here, *k*_*x*_ and *k*_*z*_ are in-plane and vertical wavevector components of a resonance mode in the nominal cavity layer with a thickness of *t*_*c*_, respectively. The phase *ϕ*_*i*_ is the reflectivity phase from the *i*-th mirror, as shown in Fig. 1(b). Note that the reflection phases, *ϕ*_1_ and *ϕ*_2_ depend on the in-plane wavevector *k*_*x*_. The VCS modes of interest typically have a lateral extension of several times of wavelength, which corresponds to the reciprocal-space mode profiles distributing mostly in the vicinity of *k*_*x*_ = 0 (or small angle of incident *θ*). Thus, the dispersion is obtained by Taylor expanding all parameters in the expression *ψ* = 2*mπ* close to the Γ-point where *k*_*x*_ = 0 (details in Supplementary Information)^25^:

1 $$\begin{array}{ccc}\omega  & = & {\omega }_{0}+\displaystyle \frac{1}{2}{\beta }_{x}{k}_{x}^{2},\\ {\beta }_{x} & \triangleq  & \displaystyle \frac{c}{2{n}_{c}}\displaystyle \frac{1}{{t}_{{\rm{e}}{\rm{f}}{\rm{f}}}}(\displaystyle \frac{2c{t}_{c}}{{n}_{c}{\omega }_{0}}+\displaystyle \frac{{{\rm{\partial }}}^{2}{\varphi }_{1}}{{\rm{\partial }}{k}_{x}^{2}}+\displaystyle \frac{{{\rm{\partial }}}^{2}{\varphi }_{2}}{{\rm{\partial }}{k}_{x}^{2}}),\end{array}$$

where *ω*_0_ is the resonance frequency at normal incidence, *c* is the speed of light in vacuum, *t*_eff_ ($\triangleq$ *t*_1_ + *t*_c_ + *t*_2_) is the effective cavity thickness, *t*_*i*_ $(\triangleq -\,\tfrac{c}{2{n}_{c}}\partial {\varphi }_{i}/\partial \omega )$ is the phase penetration into the *i*-th mirror, and the derivatives ${\partial }^{2}{\varphi }_{i}/\partial {k}_{x}^{2}$ are evaluated at *k*_*x*_ = 0. The parameter ${\beta }_{x}(\,=\,{\partial }^{2}\omega /\partial {k}_{x}^{2})$ represents the cavity dispersion curvature along the *x*-direction. It consists of three terms; the first term results from the round-trip propagation in the nominal cavity and depends on its thickness *t*_*c*_; the second and third terms account for mirror contributions and depend on the angular response of the reflectivity phase $({{\rm{\partial }}}^{2}{\varphi }_{i}/{\rm{\partial }}{k}_{x}^{2})$. When a HCG reflector is employed in VCS, it is possible to engineer the corresponding mirror contribution term to have a positive, negative, or even zero value^25^. Therefore, both the sign and magnitude of the dispersion curvature, *β*_*x*_ or equivalently photon’s effective mass, *m*_*x*_ ($\hslash /{m}_{x}={\partial }^{2}\omega /\partial {k}_{x}^{2}={\beta }_{x}$ where $\hslash$ is the reduced Planck constant) of a mode in a VCS can be engineered by using a HCG reflector^13, 25, 29^.

### Two coupled vertical cavities dispersion

A system of two coupled cavities is formed by three reflectors as illustrated in Fig. 1(b). Here, a HCG reflector is employed as a coupling mirror shared by two cavities. For simplicity, it is assumed that two cavities are identical, i.e. mirrors *m*_3_ and *m*_2_ are identical and both cavities have the same nominal cavity length, *t*_*c*_. The resonance frequencies of the coupled VCS can be determined from the transmissivity maximum conditions (details in Supplementary Information):

2 $$\cos \,(\psi )=\,\cos \,({\varphi }_{1}+{\varphi }_{2}-2{k}_{z}{t}_{c})\approx {r}_{1},$$

where *ψ* is the round-trip phase of a single cavity and other parameters are defined in Fig. 1(b). Here, the reflectivity amplitudes of outer mirrors, *r*_2_ and *r*_3_ are assumed to be close to unity, i.e. *r*_2_ = *r*_3_ ≈ 1. The resonance frequencies of the two hybridized modes at normal incidence, *ω*_−,0_ and *ω*_+,0_ are red- and blue-shifted by Δ*ω*_0_ with respect to the single cavity frequency *ω*_0_, respectively. They are found as ${\omega }_{\pm \mathrm{,0}}={\omega }_{0}\pm {\rm{\Delta }}{\omega }_{0}\simeq {\omega }_{0}\pm c/\mathrm{(2}{n}_{c}{t}_{{\rm{eff}}})f$, where $f\triangleq \sqrt{2(1-{r}_{1})}$, provided that *r*_1_ is also close to unity. Exemplary mode profiles of these two hybridized modes are provided in Supplementary Information.

Similar to the single VCS case, the mode dispersion can be obtained by Taylor expanding Eq. (2) close to the Γ-point (details in Supplementary Information):

3 $$\begin{array}{c}{\omega }_{\pm }={\omega }_{\pm ,0}+\displaystyle \frac{1}{2}{\beta }_{\pm ,x}{k}_{x}^{2},\\ {\beta }_{\pm ,x}\triangleq \displaystyle \frac{c}{2{n}_{c}}\displaystyle \frac{1}{{t}_{{\rm{e}}{\rm{f}}{\rm{f}}}}(\displaystyle \frac{2c{t}_{c}}{{n}_{c}{\omega }_{0}}+\displaystyle \frac{{{\rm{\partial }}}^{2}{\varphi }_{1}}{{\rm{\partial }}{k}_{x}^{2}}+\displaystyle \frac{{{\rm{\partial }}}^{2}{\varphi }_{2}}{{\rm{\partial }}{k}_{x}^{2}}\mp \displaystyle \frac{1}{f}\displaystyle \frac{{{\rm{\partial }}}^{2}{r}_{1}}{{\rm{\partial }}{k}_{x}^{2}})={\beta }_{x}\pm {\alpha }_{x},{\rm{w}}{\rm{h}}{\rm{e}}{\rm{r}}{\rm{e}}{\alpha }_{x}\triangleq -\displaystyle \frac{c}{2{n}_{c}{t}_{{\rm{e}}{\rm{f}}{\rm{f}}}f}\displaystyle \frac{{{\rm{\partial }}}^{2}{r}_{1}}{{\rm{\partial }}{k}_{x}^{2}},\end{array}$$

where all the derivatives are found at *k*_*x*_ = 0. The dispersion curvatures of the two modes in the coupled cavities, *β*_±,*x*_ are similar to that of a single cavity mode, *β*_*x*_ in Eq. (1), except for the term, *α*_*x*_. This term accounts for the effect of coupling on dispersion. We need to note that this coupling term depends on the reflectivity amplitude, *r*_1_ and its second derivative of the shared mirror, *m*_1_, not on the reflectivity phase, *ϕ*_1_ or its derivatives.

**Figure 2:**
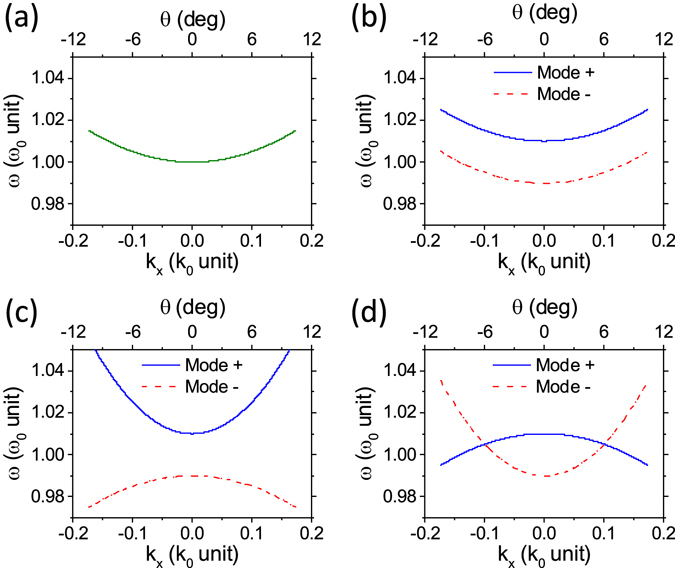
(**a**) The dispersion characteristic of a single VCS with *β*_*x*_ = *c* ^2^/*ω*_0_. (**b**–**d**) Characteristic dispersions of coupled VCSs when (**b**) ${\alpha }_{x}\ll {\beta }_{x}$, (**c**) *α*_*x*_ = +2*β*_*x*_, and (**d**) *α*_*x*_ = −2*β*_*x*_. Here, Δ*ω*_0_ is assumed to be 0.01*ω*_0_. In graphs, frequency, *ω* and wavevector, *k*_*x*_ are normalized with respect to *ω*_0_ and *k*_0_ ($\triangleq$ *ω*_0_/*c*), respectively, and the Modes + and − denote the blue- and red-shifted mode, respectively.

Depending on the sign and magnitude of the coupling term, *α*_*x*_, the dispersion characteristics of the coupled system can be classified into three cases, as illustrated in Fig. 2(b,c). If $|{\alpha }_{x}|\ll |{\beta }_{x}|$ as in Fig. 2(b), the dispersion curvatures of the two hybridized modes, *β*_±,*x*_ are identical to each other. As a result, the dispersion curves of the two hybridized modes are just two replicas of the single VCS curve (c.f. Fig. 2(a)) shifted in frequency. This case occurs when the reflectivity amplitude of the shared mirror, *m*_1_ has negligible angular dependence, for example as in metallic mirrors and DBRs. On the other hand, if |*α*_*x*_| is comparable or larger than |*β*_*x*_|, as shown in Fig. 2(c,d), *β*_+,*x*_ and *β*_−,*x*_ are different from each other as well as significantly differing from the single VCS value, *β*_*x*_. Particularly, the case of Fig. 2(d) seems interesting, since there is a possibility of obtaining a Dirac-cone like point in dispersion characteristics close to the Γ-point, which will be discussed elsewhere. Thus, the dispersion property of the coupled VCS can be engineered to a larger extent than a single VCS case due to the coupling term in the dispersion curvature. Furthermore, as it is shown below, the additional reflector can provide new possibilities for the coupled VCS, which are not achievable for the single VCS, such as designing a mode with a large dispersion curvature while keeping Q-factor large, and dynamical tuning of dispersion curvature.

### Large dispersion curvature while keeping Q-factor large

The Q-factor of a mode in the single VCS, formed just by the two mirrors *m*_1_ and *m*_2_ (c.f. Fig. 1(b)), is determined from *Q* = −2*πn*_*c*_ *t*_eff_/[*λ*_0_ log (*r*_1_ *r*_2_)], where *λ*_0_ is the resonance wavelength of the cavity in vacuum (details in Supplementary Information). Since the mirror reflectivity amplitudes, *r*_*i*_ depends on in-plane wavevector, *k*_*x*_, the Q-factor also is a function of *k*_*x*_. For many applications of vertical cavities, the mode Q-factor is required to be larger than a minimum value over a range of *k*_*x*_ values corresponding to small incident angles, especially for devices with a small lateral size. For instance, the Q-factor of small-aperture VCSELs should be as high as a few thousands over an incident angle of several degrees, to reach lasing condition with known gain materials^30^. Similar criterion applies also to a polariton laser^13^. For conventional reflectors such as DBRs or metallic mirrors, the reflectivity amplitude drops slowly with the incident angle increasing, while it may vary considerably for HCGs^30^. Thus, it could be difficult to obtain both a large dispersion curvature and a high Q-factor from a HCG-based single VCS with a small lateral mode size.

**Figure 3:**
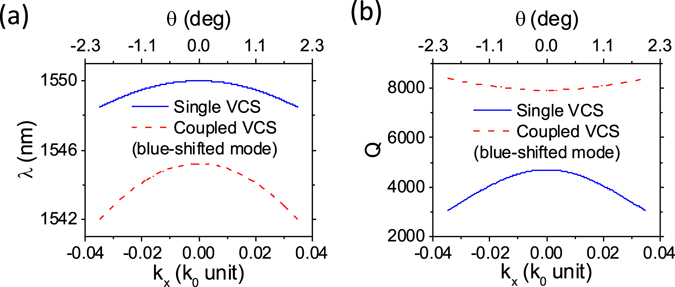
(**a**) The resonance wavelength *λ*, and (**b**) the Q-factor versus the in-plane wavevector component in the *x*-direction *k*_*x*_ (or incident angle *θ*), for a reference mode in single VCS (blue) and the blue-shifted mode in structure Fig. 1 (red). The dispersion curvature is 125 and 270 m^2^/s for the single and coupled VCS case, respectively. The Q-factor of the coupled cavity mode has larger values, which remains large for small *k*_*x*_ values, while that of the single cavity mode drops rapidly. Simulation details are provided in Supplementary Information.

This restriction can be removed in the coupled VCS. In the structure of Fig. 1(b), the Q-factor of the two hybridized modes depend mainly on the reflectivity amplitudes of outer mirrors, *m*_2_ and *m*_3_, since the Q-factor is determined by the rate at which a photon escapes from the coupled VCS through the outer mirrors. Thus, it is possible to engineer the dispersion related terms, ${\partial }^{2}{\varphi }_{1}/\partial {k}_{x}^{2}$ and ${\partial }^{2}{r}_{1}/\partial {k}_{x}^{2}$ in Eq. (3) without a strict constraint, which are related to the inner HCG, *m*_1_. For instance, the resonance wavelength and Q-factor versus in-plane wavevector *k*_*x*_ of a single VCS mode and a blue-shifted mode in a coupled VCS are shown in Fig. 3(a,b), respectively. Both designs use the same HCG parameters. The dispersion curvature of the blue-shifted mode in the coupled VCS is twice larger than that of the single VCS mode, which is due to the coupling term in the dispersion expression. Furthermore, its Q-factor is larger and remains large for the small *k*_*x*_ values, compared to the single VCS case.

### Dynamical tuning of dispersion curvature

For experimental realization, a coupled VCS structure is proposed, based on the wafer-bonding technique^26, 31^ and the hybrid grating (HG) reflector^32^. Using this design, it is numerically shown that in a coupled VCS the dispersion curvature can be dynamically tuned to a large extent, while the resonance frequency being kept constant.

**Figure 4:**
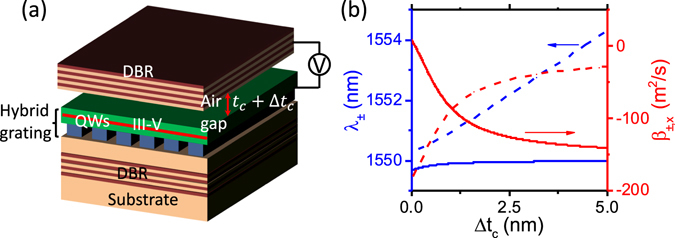
(**a**) Schematic view of a system of two coupled cavities consists of two outer DBRs and a hybrid grating (HG) reflector as the common mirror. (**b**) The resonance wavelength of the cavity modes *λ*_±_ (blue) and the mode dispersion curvatures in the *x*-direction *β*_±,*x*_ (red) as a function of airgap thickness detuning Δ*t*_*c*_ for the coupled cavities of Fig. 4(a). Simulation details are provided in Supplementary Information.

Let us describe the proposed structure as well as discussing its fabrication feasibility. As shown in Fig. 4(a), the proposed structure consists of two DBRs as outer mirrors and a HG as a coupling mirror. The HG, which is composed of a Si grating layer and a cap layer made of III-V semiconductors, is a variant of HCG^16, 32, 33^. The reflection properties of HGs are similar to those of HCGs^16^. The lower cavity is made of SiO_2_ and has a fixed thickness of *t*_*c*_. The upper cavity is made of air. Its thickness can be varied to *t*_*c*_ + Δ*t*_*c*_ by introducing an electrostatic force between the top DBR and the cap layer^34^. The bottom Si/SiO_2_ DBR, the SiO_2_ lower cavity layer, and the Si grating layer can be formed by using standard dielectric deposition technique, e-beam lithography, and dry etching process. The cap layer, a sacrificial layer for the upper air cavity, and the top DBR, all made of III-V semiconductors are prepared by III-V epitaxy growth. Then, the III-V part is wafer-bonded onto the Si grating layer. Afterward, the III-V substrate is removed, mesa structures are made, and the top DBR is membranized by removing the sacrificial layer. Finally, metal contacts are formed. The wafer-bonding can be feasibly done with a high yield in several ways, e.g., direct wafer-bonding as we did for hybrid Si-on-chip lasers and photodetectors^26, 31, 33^, or transfer-printing process^35, 36^. It is noted that the field strength within the cap layer can be very strong. Thus, the optical gain sufficient for lasing can be generated, provided that the cap layer includes a gain material^33^. In this work, however, it is assumed that the cap layer does not include a gain material, focusing solely on the tuning properties. The gain material can be easily modeled by introducing an imaginary refractive index.

Figure 4(b) shows the mode resonance wavelength and its dispersion curvature as a function of the upper cavity thickness change, Δ*t*_*c*_. The dispersion curvature of the two hybridized modes in the coupled cavity system are changed dramatically (from −140 to +10 *m* ^2^/*s* for the blue-shifted mode and from −185 to −28 *m* ^2^/*s* for the red-shifted mode). For comparison, in a single VCS case without the bottom DBR, the change in dispersion curvature is very small (just from −24 to −32 *m* ^2^/*s*). Furthermore, for the mode in single VCS, the resonance wavelength varies considerably when the cavity length is tuned (more than 4 nm in the current case). But, the photon’s energy (or equivalently resonance wavelength) of the blue-shifted mode in coupled VCS is approximately constant, while its effective mass (or equivalently dispersion curvature) changes dramatically. Therefore, one can tune the photon’s effective mass dynamically, either its sign or value, while keeping its energy approximately constant, in a coupled vertical cavity system. The tuning speed of the photon’s effective mass depends mainly on the speed of top DBR mechnical movement in this structure. By employing an electrostatic force between the top DBR and the cap layer, the photon’s effective mass can be modulated at a speed of hundreds of kHz^37^. Furthermore, by replacing the top DBR with a HCG mirror, it can be further enhanced to MHz range thanks to the lighter mass of HCG mirrors than that of DBRs^38^.

## Discussion

The ability to dynamically modify the photon’s effective mass while keeping its energy constant, may open a door for novel applications. For instance, in a polariton laser, it can result in modifying directly the polariton mass by changing the photon’s effective mass, and consequently control the dynamics and condensate formation. In a conventional vertical cavity laser, one can modify the spontaneous emission rate, which change the linewidth of the laser output. Furthermore, if an in-plane heterostructure is formed in this structure, changing the photon’s effective mass can dramatically vary the properties of heterostructure^25^. Finally, the possibility of modulating the photon’s effective mass by introducing an electrical contact in the structure seems very interesting for investigating the fundamental of light-matter interactions, and novel applications can be expected. For instance, since changing the dispersion curvature modify the DOS, one can tune the Purcell enhancement factor, and consequently the spontaneous emission rate of an emitter^24^. It should be emphasized that modifying the dispersion property dynamically is possible in other ways, e.g. by applying a magnetic field or employing electrorefractive effects. However, a complex system is required for these approaches, and the modification is usually smaller compared to the approach proposed here, since these effects are relatively weaker.

In conclusion, we have shown that the dispersion characteristics of coupled vertical cavity structures employing a HCG as a coupling mirror can be fully controlled by engineering the angular dependence of the reflection amplitude of the coupling mirror. Three distinct types of dispersion characteristics can be obtained, depending on the angular dependence of the HCG reflectivity amplitude. As an important feature, the mode dispersion curvature can be designed to attain a large value as well as retaining its large Q-factor. Furthermore, it is shown that the mode dispersion curvature can be tuned dynamically to a large extent while the mode frequency is maintained nearly constant. Thus, the coupled vertical cavity structure based on a HCG may extend the design possibilities for engineering the dispersion property of an optical cavity, which has a great importance for cavity quantum electrodynamics studies and polariton laser applications.

## Methods

### Simulation

For numerical simulations, an in-house developed simulator based on the rigorous coupled wave analysis (RCWA) method^39, 40^, also referred to as Fourier modal method (FMM), is employed. The mode dispersion and Q-factor calculations are performed using the approach explained in ref. 41. The mirrors *m*_2_ and *m*_3_ are implemented as 3.5-pair Si/SiO_2_ DBRs, while *m*_1_ is implemented as a HCG or HG. The HCG is a single Si grating layer and the HG consists of an InP cap layer and a Si grating layer^16, 28^. It is assumed that the input and output media, are infinite half spaces, and a 0.5*λ*-long cavity is designed for the telecommunication wavelength of 1550 nm. In all simulations, transverse magnetic (TM) polarized light, i.e. electric field perpendicular to the grating bars is considered. Similar results can be obtained for transverse electric (TE) polarized light by changing design parameters. The layer thicknesses and refractive indices of the simulated structures are provided in the Supplementary Information.

## Electronic supplementary material

supplementary material
