# Supplementary material for: Dynamical dispersion engineering in coupled vertical cavities employing a high-contrast grating
Source: Sci Rep. 2017 May 18;7:2123. doi: 10.1038/s41598-017-02394-9 (PMC5437060; doi:10.1038/s41598-017-02394-9)
Supplement: Supplementary file 1 — supplementary material [file 41598_2017_2394_MOESM1_ESM.pdf]

Supplementary Information

Dynamical dispersion engineering in coupled vertical cavities employing a high-contrast grating

Alireza Taghizadeh and Il-Sug Chung*

Department of Photonics Engineering (DTU Fotonik),

Technical University of Denmark, DK-2800 Kgs. Lyngby, Denmark.

Abstract

This document provides supplementary information to Dynamical dispersion engineering in coupled vertical cavities employing a high-contrast grating." The detailed derivation of all equations in the article and the simulation parameters used for generating numerical results are provided here.

* e-mail: ilch@fotonik.dtu.dk

I. DERIVATION OF EQUATIONS

In this section, the detailed derivation of all equations in the main article is provided. The vertical direction, the in-plane direction perpendicular to grating bars, and the in-plane direction parallel to grating bars are represented by the z-, x-, and y-directions, respectively [c.f. Fig. 1]. In the main article, the dispersion properties are discussed for the x-direction. However, it is noteworthy that similar results can be obtained for the y-direction as well.

A. Single Vertical Cavity

For a single vertical cavity structure (VCS) formed by two mirrors with the reflectivity amplitudes, r_1 and r_2 and reflectivity phases, ϕ_1 and ϕ_2, the transmissivity, T is obtained by using the transfer matrix method as [1]:

$$T = \frac{(1 - r_1^2)(1 - r_2^2)}{1 + r_1^2 r_2^2 - 2r_1 r_2 \cos(\psi)} \tag{S1}$$

where ψ is the round trip phase in the cavity, which is given by

$$\psi \triangleq \phi_1(\omega, k_x) + \phi_2(\omega, k_x) - 2k_z t_c. \tag{S2}$$

Here, k_x and k_z, and ω are the wavevector components and angular frequency of a plane wave in the nominal cavity layer with thickness, t_c and refractive index, n_c. Assuming $k_x \ll k_z$,

$$k_z = \left[\left(\frac{\omega n_c}{c}\right)^2 - k_x^2\right]^{1/2} \simeq \left(\frac{\omega n_c}{c}\right)\left[1 - \frac{1}{2}\left(\frac{c}{\omega n_c}\right)^2 k_x^2\right], \tag{S3}$$

where c is the speed of light in vacuum. Resonance occurs at frequencies, ω at which T is locally maximized. The maximum condition is satisfied when:

$$\psi = 2m\pi, \tag{S4}$$

which is a constructive interference condition for the round-trip phase. Then, the resonance frequency at normal incidence ($k_x = 0$), ω_0 is determined by:

$$\frac{2\omega_0 n_c t_c}{c} = -2m\pi + \phi_{1,0} + \phi_{2,0}, \tag{S5}$$

where $\phi_{i,0} \triangleq \phi_i(\omega_0, 0)$.

The quality-factor (Q-factor) of a resonance mode with a central frequency, ω_0 and a full-width half-maximum (FWHM) width, $\Delta\omega_{\rm FWHM}$ is determined by:

$$Q = \frac{\omega_0}{\Delta\omega_{\rm FWHM}}, \tag{S6}$$

where $\Delta\omega_{\rm FWHM}$ can be obtained as:

$$\Delta\omega_{\rm FWHM} = \left(\frac{\partial\psi}{\partial\omega}\right)^{-1}\Delta\psi_{\rm FWHM} = \left(\frac{2n_c}{c}t_{\rm eff}\right)^{-1}\Delta\psi_{\rm FWHM}. \tag{S7}$$

Here, the effective cavity thickness, $t_{\rm eff}$ and the FWHM width of the roundtrip phase, $\Delta\psi_{\rm FWHM}$ are given by:

$$t_{\rm eff} \triangleq t_c - \frac{c}{2n_c}\frac{\partial\phi_1}{\partial\omega} - \frac{c}{2n_c}\frac{\partial\phi_2}{\partial\omega} \tag{S8}$$

and

$$\Delta\psi_{\rm FWHM} = 2\cos^{-1}\left(\frac{2r_1r_2}{1+r_1^2r_2^2}\right) \simeq 2(1-r_1r_2) \simeq -2\ln(r_1r_2), \tag{S9}$$

where it is assumed that both r_1 and r_2 are close to unity [2]. By inserting Eqs. (S7) and (S9) into Eq. (S6), the Q-factor of a resonance mode is expressed by:

$$Q \simeq -\frac{2\pi n_c t_{\rm eff}}{\lambda_0}\frac{1}{\ln(r_1r_2)}, \tag{S10}$$

where $\lambda_0 \triangleq 2\pi c/\omega_0$.

Since a resonance mode has a finite Q-factor and a finite lateral mode size, it has finite distributions of ω and k_x around $\omega = \omega_0$ and $k_x = 0$, respectively. Around these, the reflectivity phases, ϕ_i can be Taylor-expanded, keeping the first non-zero derivatives with respect to k_x and ω [3]:

$$\phi_i(\omega, k_x) \simeq \phi_{i,0} + \frac{1}{2}\frac{\partial^2\phi_i}{\partial k_x^2}k_x^2 + \frac{\partial\phi_i}{\partial\omega}\Delta\omega, \tag{S11}$$

where $\Delta\omega = \omega - \omega_0$ and the derivatives are evaluated at $(\omega, k_x) = (\omega_0, 0)$. Inserting Eqs. (S3) and (S11) into Eq. (S4) leads to Eq. (1):

B. Two Coupled Vertical Cavities

For a system of two identical vertical cavities coupled through a common reflector, as illustrated in Fig. 1(b), the transmissivity, T can be obtained by using the transfer matrix

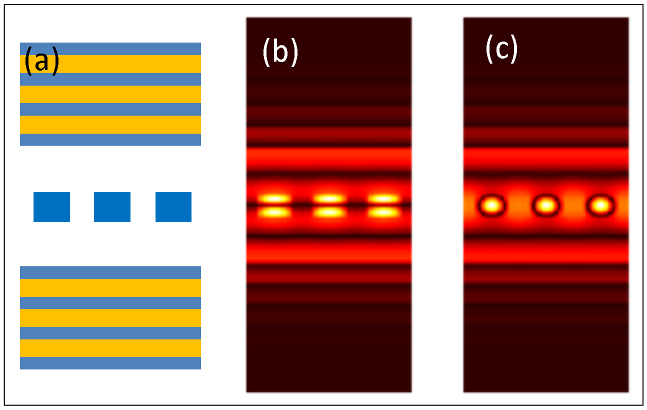

Supplementary Figure 1. (a) Schematic cross-section of a system of two coupled vertical cavities employing an HCG reflector between two DBRs. (b), (c) A typical field profile ($|H_y|$) of the blue-shifted and red-shifted modes of the structure in (a).

method[1]:

$$T = \frac{(1-r_1^2)(1-r_2^2)^2}{1+r_2^4+4r_1^2r_2^2-4r_1r_2(1+r_2^2)\cos(\psi)+2r_2^2\cos(2\psi)}, \tag{S12}$$

where ψ is the round-trip phase of each cavity, r_1 is the reflectivity amplitude of the common mirror, and r_2 is the reflectivity amplitude of two outer mirrors. Resonance occurs at a condition that T is locally maximized, which is given by:

$$\cos(\psi) = \frac{r_1(1+r_2^2)}{2r_2} \simeq r_1, \tag{S13}$$

which is Eq. (2). In the last equality, it is assumed that $r_2 \approx 1$.

At this resonance condition, two hybridized modes are formed, which are a linear combination of the two modes of the individual cavities. Exemplary field profiles of the two hybridized modes are shown in Fig. **1**. The frequencies of two hybridized modes, ω_- and ω_+ are red-shifted and blue-shifted with respect to the mode of single cavity, ω_0, respectively:

$$\omega_{\pm,0} \simeq \omega_0 \pm \frac{c}{2n_c t_{\text{eff}}} f \quad \text{where} \quad f \triangleq \sqrt{2(1-r_1)}. \tag{S14}$$

Eq. (S13) can be Taylor-expanded around $k_x = 0$ and $\omega = \omega_{\pm,0}$, assuming Eq. (S13) is valid around this point. For this, ϕ_i and r_1 are expanded while keeping the first non-zero derivatives with respect to k_x and ω:

$$\begin{aligned} \phi_i(\omega, k_x) &\simeq \phi_{i,0} + \frac{1}{2}\frac{\partial^2 \phi_i}{\partial k_x^2} k_x^2 + \frac{\partial \phi_i}{\partial \omega}\Delta\omega, \\ r_1(\omega, k_x) &\simeq r_{1,0} + \frac{1}{2}\frac{\partial^2 r_1}{\partial k_x^2} k_x^2 + \frac{\partial r_1}{\partial \omega}\Delta\omega, \end{aligned} \tag{S15}$$

where $r_{1,0} \triangleq r_1(\omega_0, 0)$, and the derivatives are evaluated at $(\omega, k_x) = (\omega_0, 0)$. Usually, the last term in the $r_1(\omega, k_x)$ is much smaller than the second term and it is neglected here. Inserting the expressions in Eq. (S15) in to the last equality of Eq. (S13) and using Eq. (S14), Eq. (3) is obtained for the dispersion of two hybridized modes.

II. SIMULATION PARAMETERS

In the following tables, the layer thicknesses and refractive indices of the simulated structures are provided.

No.	Layer	Refractive index	Thickness (nm)	Comment
1	Superstrate	1.0	∞	Infinite half space
2, 3	DBR-h<br>DBR-l	3.48<br>1.48	111.4<br>261.8	3.5-pairs top DBR
4	Cavity	1.0	617.5	Top cavity
5	Grating	3.48/1.0	450	Period: 750 nm, Bar Width: 502.5 nm
6	Cavity	1.0	617.5	Bottom cavity
7, 8	DBR-h<br>DBR-l	3.48<br>1.48	111.4<br>261.8	3.5-pairs bottom DBR
9	Substrate	1.0	∞	Infinite half space

Supplementary Table 1: Structure dimensions and refractive indices related to Fig. 3, for the blue-shifted mode in coupled cavity system. For the mode of single cavity, the bottom cavity and DBR are removed.

[1] Z. Knittl, *Optics of thin films: an optical multilayer theory* (Wiley London:, 1976).

[2] A. Taghizadeh, J. Mørk, and I.-S. Chung, J. Lightwave Technol. **34**, 4240 (2016).

[3] A. Taghizadeh, J. Mørk, and I. S. Chung, Appl. Phys. Lett. **107**, 181107 (2015).

No.	Layer	Refractive index	Thickness (nm)	Comment
1	Superstrate	1.0	∞	Infinite half space
2, 3	DBR-h<br>DBR-l	3.48<br>1.48	111.4<br>261.8	3.5-pairs top DBR
4	Cavity	1.0	$801.7+\Delta t_c$	Tunable air-gap
5	Cap	3.1661	340	InP cap-layer
6	Grating	3.48/1.0	500	Period: 790 nm, Bar Width: 442.5 nm
7	Cavity	1.48	770	Bottom cavity
8, 9	DBR-h<br>DBR-l	3.48<br>1.48	111.4<br>261.8	3.5-pairs bottom DBR
10	Substrate	1.48	∞	Infinite half space

Supplementary Table 2: Structure dimensions and refractive indices related to Fig. 4. The airgap thickness varies by changing Δt_c from 0 to 5 nm. For the reference single cavity, the bottom cavity and DBR are removed.
